# Exploring Personality Features in Patients with Affective Disorders and History of Suicide Attempts: A Comparative Study with Their Parents and Control Subjects

**DOI:** 10.1155/2014/291802

**Published:** 2014-03-02

**Authors:** Beatriz Camarena, Ana Fresán, Emmanuel Sarmiento

**Affiliations:** ^1^Subdirección de Investigaciones Clínicas, Genética Psiquiátrica, Instituto Nacional de Psiquiatría Ramón de la Fuente, Calz. México-Xochimilco 101, 14370 Mexico City, Mexico; ^2^Subdirección de Investigaciones Clínicas, Epidemiología Psiquiátrica, Instituto Nacional de Psiquiatría Ramón de la Fuente, Calz. México-Xochimilco 101, 14370 Mexico City, Mexico; ^3^Hospital Psiquiátrico Infantil Juan N. Navarro, San Buenaventura 86, Col. Belisario Dominguez, 14080 Mexico City, Mexico

## Abstract

Personality traits are important candidate predictors of suicidal behavior. Several studies have reported an association between personality/temperament traits and suicidal behavior, suggesting personality traits as intermediary phenotypes related to suicidal behavior. Thus, it is possible that suicide attempts can be accounted for by increased familial rates of risk personality traits. The aim of this work was to evaluate personality traits in affective disorder patients with attempted suicide and to compare them with the personality trait scores of their parents. In addition, ITC scores in the two groups were compared with a healthy control sample. The patients evaluated met the DSM-IV criteria for major depression disorder or dysthymia and had a documented history of suicide attempts. Psychiatric diagnoses of patients and parents were done according to the SCID-I and the personality was assessed using the Temperament and Character Inventory. We analyzed 49 suicide attempt subjects and their parents (*n* = 95) and 89 control subjects. We observed that temperament and character dimensions were similar between patients and their parents (*P* > 0.05). In particular, we observed that high HA and low P, SD, and CO were shared among families. Our study is the first to report that the personality traits of affective disorder patients with a history of attempted suicide are shared between patients and their parents.

## 1. Introduction

Suicidal behavior is a major health problem worldwide, particularly associated with affective disorders [1, 2]. The World Health Organization reported that suicide accounts for 2% of the world's deaths. Mexico, in particular, reported that between 2.5 and 4.3% of its population attempted suicide at some point in their life [3].

A familial transmission study showed an increased risk of suicide attempt in the offspring of suicide attempters with affective disorders [4]. Family, adoption, and twin studies support the theory that genetic factors are involved in suicidal behavior [4–7]. However, environmental factors could also be involved in the development of a complex phenotype such as suicidal behavior. In particular, the familial transmission of an adverse family environment, imitation, or bereavement could be related to heritable risk factors [8]. Interestingly, it was reported that suicide ideation and attempts were strongly associated with parental psychopathology [9, 10]. In addition, it has been suggested that certain personality traits may predispose to affective disorders and suicidal behavior [11]. Personality traits are important candidate predictors of suicidal behavior because personality represents the pervasive attitude of an individual towards the environment [12]. Several studies have reported an association between personality/temperament traits and suicidal behavior [13, 14]. In addition, personality traits have been suggested as intermediary phenotypes related with suicidal behavior [15, 16]. In particular, high novelty seeking and harm avoidance have been commonly observed in attempted suicide subjects [13, 17–20]. Moreover, Calati et al. [20] reported lower scores of self-directedness and cooperativeness compared with control subjects.

Gureje et al. [9] suggested that the use of psychological traits should incorporate new information about the risk of suicidal behavior. It is possible that suicide attempts can be accounted for by increased familial rates of risk personality traits.

The aim of the present study was to evaluate, using TCI scores, the personality traits of affective disorder patients with attempted suicide and to compare them with the personality trait scores of their parents. In addition, the TCI scores of patients and their parents were compared with a healthy control sample.

## 2. Methods

### 2.1. Sample

The sample included 49 patients and 95 parents who were consecutively recruited at the outpatient services of a highly specialized mental health institution in Mexico City. The parents were recruited for a family-based association study of serotoninergic candidate genes. Patients were included if they were over 18 years of age, met DSM-IV criteria for major depression disorder or dysthymia [21], had a documented history of suicide attempts, and at least one of their parents voluntarily agreed to participate in the study. Patients were excluded if they had any concomitant medical or neurological illness, current substance abuse or dependence, with the exception of nicotine, or were agitated. Patients were under pharmacological treatment and clinically stable according to the discretion of the treating psychiatrist.

The information about previous suicide attempts in patients was collected from medical records and a face-to-face interview with the patients and their relatives. Suicide attempt was defined as intentional self-harm that required medical treatment in an emergency or intensive care unit. The information concerning the history of affective disorders or suicide attempts in parents was obtained through clinical interviews with them. This information was recorded in a previously designed form. Psychiatric diagnoses of patients and parents were done according to the Structured Clinical Interview for DSM-IV Axis I Disorders (SCID-I) [22]. All subjects gave written informed consent to participate in the study. The Ethics Review Board of the mental health institution approved the study.

The control group comprised 89 unrelated individuals recruited from the general population in Mexico City at several public locations. The subjects who agreed to participate were initially screened for psychopathology using the Symptoms Checklist 90 (SCL-90) [23, 24] and were questioned about any previous suicide attempt. The subjects who had a score higher than 2 in any of the SCL-90 subscales or reported any previous suicide attempt were excluded. An appointment was fixed in the mental health institution to perform a clinical interview using the Structured Clinical Interview for DSM-IV (SCID-I) [22]. The subjects who met DSM-IV criteria for any Axis I diagnosis were also excluded.

Personality was assessed using the Temperament and Character Inventory (TCI) [25, 26], a 240-question self-report instrument with a true-false format. The psychometric properties of the TCI were tested for their use in the Mexican population, providing adequate reliability and validity values [27]. The TCI includes seven dimensions, four of temperament and three of character. The temperamental dimensions are considered partially heritable responses to experiences that are constant throughout life; they are termed novelty seeking (NS), harm avoidance (HA), reward dependence (RD), and persistence (P). The character dimensions refer to individual differences in goals and values which develop by insight learning through personal and social experience; they are self-directedness (SD), cooperativeness (CO), and self-transcendence (ST) [28].

### 2.2. Statistical Analysis

The information about suicide attempts and demographic and clinical features was described with frequencies and percentages for categorical variables and with means and standard deviations (S.D.) for continuous variables.

The Kolmogorov-Smirnov test was used for testing the normal distribution of the TCI dimensions in each group. The comparison of the TCI dimensions between control subjects, patients, and parents was done with univariate ANOVA tests. The significance level for the tests was established at *α* ≤ 0.05. If a significant difference was detected in an ANOVA test, a Bonferroni correction was performed. The significance level of Bonferroni correction was established at *P* < 0.016 (0.05/3 groups). As gender, age, and level of education may influence personality traits, an additional ANCOVA model was performed including these variables as covariates and the group as the fixed factor. All analyses were carried out using the SPSS statistical software, version 20.0.

The statistical power was calculated by the program G*Power 3.1 for a *post hoc* analysis with *α* = 0.05 to detect the medium effect size of *d* = 0.5 according to Cohen's definitions; it showed a statistical power of 0.88 [29].

## 3. Results

The demographic and clinical characteristics of the sample are shown in Table 1.

The Kolmogorov-Smirnov test showed a normal distribution of the TCI scores in each group, with *P* values > 0.05 (control group range: 0.67 to 1.26; patient group range: 0.62 to 1.06, and parents group range: 0.76 to 1.18).

The mean scores and standard deviations of the temperament and character dimensions of each group are reported in Table 2. Differences were found between groups in all personality dimensions, with the exception of the character dimension ST.

In post hoc comparisons, it was observed that temperament and character dimensions were similar between patients and their parents (all *P* values > 0.05). Significant differences emerged when these two groups were compared with the control subjects, which are also shown in the post hoc comparisons in Table 2. The patients exhibited higher scores on the temperament dimensions of HA and NS and lower scores in the character dimensions SD and CO, compared with controls. The parents showed higher scores in HA and lower scores in RD, P, SD, and CO, compared with the control subjects.

The ANCOVA analysis adjusted for gender showed a significant influence on the character dimensions CO (*F* = 6.57, df 1, *P* = 0.01) and ST (*F* = 5.26, df 1, *P* = 0.02), where female subjects exhibited higher scores in both dimensions. In addition, age influenced character dimension ST and the level of education influenced the temperament dimension RD (*F* = 5.82, df 1, *P* = 0.01); subjects with higher education had higher scores on RD.

## 4. Discussion

Personality traits are important risk factors that influence the vulnerability to suicidal behavior and affective disorders [15, 30]. The familial aggregation of suicide attempts is not explained only by gene transmission but also by family-environmental factors [8, 9, 31, 32].

This exploratory study is the first report to analyze the personality traits of affective disorder patients with suicide attempts and those of their parents. We replicated the findings of high harm avoidance and novelty seeking and low self-transcendence and cooperativeness in TCI scores for affective disorder patients with history of suicide attempts compared with a control sample [20, 33–38]. The parents' group showed higher HA and lower RD, P, SD, and CO scores compared with the control group. Interestingly, the comparison of TCI scores between patients and their relatives did not show statistical differences in personality traits, showing that families with affective disorders and history of suicide attempts share similar personality profiles. Therefore, our findings support the hypothesis about parental transmission of risk traits to offspring and genetic susceptibility to affective disorders and suicide attempts. Interestingly, gender, age, and level of education influenced ST, increasing the probability of overlooking an effect between the groups compared.

Family and twin studies have suggested that genetic factors are involved in suicide and affective disorders. In addition, temperamental traits such as HA are partly heritable dimensions related to abnormalities in the serotoninergic system [26]. It is possible that the findings observed in the families studied may be explained by an interaction between genetic vulnerability and family environment; however, this hypothesis should be further studied.

In our sample, 13.4% of the parents of patients with affective disorders and history of suicide attempts reported a lifetime suicide attempt, which is higher than the percentage observed in the Mexican population [3]. Also, we observed that 15% of the parents had a current depressive episode and 38.7% presented a substance abuse disorder, supporting the role of family psychopathology on the risk of suicide attempts among offspring. Borges et al. [10] did not demonstrate a correlation with parental depression and substance abuse disorder in suicide attempters in a Mexican National Survey. It is possible that parental personality profiles could be associated with an increase in the susceptibility to affective disorders and suicide attempts in offspring of families with higher rates of psychopathology.

## 5. Conclusions

In conclusion, our study is the first to compare the personality traits of patients with affective disorders and history of attempted suicide with the personality traits of their parents, showing that high HA and low P, SD, and CO are shared among families with suicide attempts.

The present study has several limitations. The families were originally recruited for a genetic study and from a specialized clinical unit; therefore, our findings cannot be extrapolated to the general population. The study has a small sample size we did not screen for axis II disorders. In addition, the control sample was not matched by gender and age; therefore, an independent group should confirm our findings with a larger sample size to obtain a final conclusion.

## Figures and Tables

**Table 1:** Demographic and clinical characteristics of the sample.

Characteristics	Suicide attempt	Parents	Controls
Gender			
Female	40 (81.6%)	46 (50.5%)	66 (74.2%)
Male	9 (18.4%)	47 (49.5%)	23 (25.8%)
Age (mean ± DS, years)	24.3 ± 6.1	51.3 ± 9.0	35.7 ± 9.5
School education (mean ± DS, years)	12.2 ± 2.3	10.8 ± 3.1	12.04 ± 2.5
Major depression disorder	38 (77.5%)	14 (15%)	0
Dysthymia	11 (22.4%)	0	0
Age of illness onset (mean ± DS, years)	20.3 ± 6.4	—	—
Previous psychiatric hospitalizations	14 (28%)	—	—
Substance abuse disorder	25 (51%)	36 (38.7%)	0
Suicidal behavior			
Number of suicide attempts (mean ± DS)	2.0 ± 1.0	1.0 ± 0.2	—
Suicide attempt method			
Medications	32 (65.3%)	14 (15%)	—
Stabbing weapons	11 (22.5%)	—	—
Hanging	6 (12.2%)	—	—

**Table 2:** Temperament and character dimensions among groups.

TCI features	Control (C) (*n* = 89)		Patients (P) (*n* = 49)		Parents (Pr) (*n* = 95)		Statistic	*Post hoc* comparisons* *P*-value	
	Mean	S.D.	Mean	S.D.	Mean	S.D.		C versus P	C versus Pr
Temperament									
Novelty seeking	19.2	5.5	22.2	5.7	19.8	6.0	*F* = 4.3, df 2, *P* = 0.01	0.01	1.00
Harm avoidance	13.6	6.6	18.8	6.1	17.0	4.7	*F* = 14.4, df 2, *P* < 0.001	<0.001	<0.001
Reward dependence	15.1	4.0	13.9	4.2	12.0	3.5	*F* = 4.4, df 2, *P* < 0.001	0.21	<0.001
Persistence	4.9	1.7	3.9	2.4	4.0	2.0	*F* = 6.1, df 2, *P* = 0.003	0.01	<0.001
Character									
Self-directedness	32.9	7.6	22.3	7.9	25.0	10.2	*F* = 28.9, df 2, *P* < 0.001	<0.001	<0.001
Cooperativeness	31.9	6.1	22.3	7.0	23.4	9.6	*F* = 35.0, df 2, *P* < 0.001	<0.001	<0.001
Self-transcendence	17.0	6.6	16.1	5.5	15.3	5.7	*F* = 1.6, df 2, *P* = 0.18	1.00	0.20

*Bonferroni correction.
